# Incorporation of Fibrin Matrix into Electrospun Membranes for Periodontal Wound Healing

**DOI:** 10.3390/bioengineering6030057

**Published:** 2019-06-30

**Authors:** Choyi Wong, Suyog Yoganarasimha, Caroline Carrico, Parthasarathy Madurantakam

**Affiliations:** 1 Department of Periodontics, School of Dentistry, Virginia Commonwealth University, Richmond, VA 23298, USA; 2 Department of Biomedical Engineering, School of Engineering, Virginia Commonwealth University, Richmond, VA 23298, USA; 3 Department of Oral Health Promotion and Community Outreach, School of Dentistry, Virginia Commonwealth University, Richmond, VA 23298, USA; 4 Department of General Practice, School of Dentistry, Virginia Commonwealth University, Richmond, VA 23298, USA

**Keywords:** electrospinning, membranes, GTR, fibrin, wound healing, regeneration

## Abstract

Guided tissue regeneration (GTR) aims to regenerate the lost attachment apparatus caused by periodontal disease through the use of a membrane. The goal of this study is to create and characterize a novel hybrid membrane that contains biologically active fibrin matrix within a synthetic polycaprolactone (PCL) electrospun membrane. Three-dimensional fibrin matrices and fibrin-incorporated electrospun membrane were created from fresh frozen plasma by centrifugation in glass vials under three different conditions: 400 g for 12 min, 1450 g for 15 min and 3000 g for 60 min. Half the membranes were crosslinked with 1% genipin. Degradation against trypsin indicated biologic stability while uniaxial tensile testing characterized mechanical properties. Continuous data was analyzed by ANOVA to detect differences between groups (*p* = 0.05). Fibrin-incorporated electrospun membranes showed statistically significant increase in mechanical properties (elastic modulus, strain at break and energy to break) compared to fibrin matrices. While crosslinking had marginal effects on mechanical properties, it did significantly increase biologic stability against trypsin (*p* < 0.0001). Lastly, membranes generated at 400 g and 1450 g were superior in mechanical properties and biologic stability compared to those generated at 3000 g. Fibrin-incorporated, crosslinked electrospun PCL membranes generated at lower centrifugation forces offers a novel strategy to generate a potentially superior membrane for GTR procedures.

## 1. Introduction

Mechanically strong biomaterials have been traditionally used to restore the structural and load-bearing function of diseased/lost tissues including periodontal defects. The minimum biological requirement for earlier implantable materials was biological inertness: the ability of the implanted material to have minimal interaction with its surrounding tissue [1]. Recent advances in materials biology and engineering has allowed the development of smart, sophisticated, surface-active materials designed to interact with their biological milieu to actively regenerate tissue [2].

Chronic periodontitis refers to the inflammation and progressive destruction of the supporting tissues of the periodontium resulting in loss of periodontal ligament and alveolar bone [3]. Restoration of the lost periodontium from periodontal disease is a paramount goal of periodontal therapy. Guided tissue regeneration (GTR) aims to regenerate the lost attachment apparatus by inducing the formation of new cementum, a new periodontal ligament and new alveolar bone through the use of a barrier membrane [4]. The principle of GTR is based on the concept of epithelial exclusion by preventing the down-growth of epithelium to allow mesenchymal stem cells from the PDL to repopulate the root surface and promote regeneration [5]. It has been suggested that these membranes must stay physically and mechanically intact for at least 4–6 weeks for regenerative therapy to be successful [6,7].

Polycaprolactone (PCL) is a biodegradable aliphatic polyester that is popular in the field of drug delivery and tissue engineering. Among its numerous advantages are the ease and relatively inexpensive manufacturing processes; versatility to make polymer blends; tunable degradation kinetics and mechanical properties as well as ease of adding functional groups to enhance biological response. [8,9] Furthermore, since the drug delivery devices made from PCL are already commercially available, developing novel biomaterials using PCL would mean ease of translation to clinic and patient care.

Biomaterial fabrication techniques aim to impart biochemical and biophysical cues by exploiting the knowledge about native tissue architecture and cell-biomaterial interactions. Electrospinning is one such versatile technique that allows generation of porous, three-dimensional scaffolds made of sub-micron to nanometer diameter fibers [10]. The process allows control over fiber alignment, diameter as well as overall scaffold architecture. Electrospun matrices have high surface area to volume ratio and are attractive in tissue engineering applications because they closely resemble native extracellular matrix [11,12,13]. Several investigators have successfully generated scaffolds from various biomaterials in combination with PCL [14,15,16,17] for a range of applications.

Fibrinogen is a soluble blood plasma protein that is converted into an insoluble polymer gel (fibrin) that is further stabilized by enzyme-mediated crosslinking to form a stable fibrin clot. In addition to providing protection against physical and chemical insults, fibrin clots also provide a blueprint for healing through its numerous cellular and molecular interactions [18,19,20]. Recent studies have explored the potential of using fibrin gels for tissue engineering purposes because of its excellent biological characteristics [21]. In spite of their excellent biological performance [22,23,24], their rapid degradation and low mechanical strength limits its clinical application.

The goal of the present study is to develop and characterize a novel natural-synthetic hybrid that retains the biological attributes of fibrin along with slow degradation and improved mechanical properties of PCL. Future studies will involve evaluating the biological performance of these hybrid membranes in vivo.

## 2. Materials and Methods

### 2.1. Electrospinning

Polycaprolactone (PCL) was dissolved in hexafluroisopropanol (HFP) at a concentration of 150 mg/mL overnight. Once completely dissolved, polymer solution was subjected to previously defined electrospinning conditions (rate: 7 mL/h, air-gap distance: 12.5 cm, voltage: 22 kV) onto a rotating mandrel revolving at 1000 rpm [25]. After electrospinning, membrane was removed from mandrel and cut into 3 cm × 1 cm uniform pieces. Membranes were treated with 38% hydrochloric acid for 1 min to improve hydrophilicity and washed with 3x PBS for five minutes each.

### 2.2. Preparation of Fibrin-Incorporated Electron Membrane

Units of fresh, never-frozen, human plasma in sodium citrate buffer were obtained from Valley Biomedical for all fibrin related experiments in this study (Winchester, VA). A volume of 4 mL of plasma (thawed to 37 °C) was reconstituted with 100 µL of 1M CaCl_2_ just prior to experiments to overcome the anticoagulant effects of citrate and to initiate clotting. Analogous to platelet rich fibrin generation, the reconstituted plasma was placed in glass vials and centrifuged at different “g” forces to create fibrin clots. In addition to making pure fibrin matrices, hybrid membranes were generated by placing electrospun membranes within the plasma prior to centrifugation. Fibrin matrices and fibrin-incorporated electrospun membranes were created at three different centrifugation conditions: 400 g for 12 min, 1450 g for 15 min and 3000 g for 60 min. Twelve replicates were created for each of the centrifugation conditions. After centrifugation, the fibrin clots and fibrin-incorporated electrospun membranes were gently removed and compressed in a platelet-rich fibrin (PRF) box (Salvin Dental Specialties, NC, USA) to make flat membranes.

### 2.3. Genipin Crosslinking of Fibrin-Incorporated Electrospun Membranes

Under sterile conditions, half of the compressed membranes created at 400 g, 1450 g and 3000 g were placed in six well culture plates and submerged in 4 mL of 1% genipin (Sigma Aldrich, St. Louis, MO, USA) for 48 h. After 48 h, membranes were washed twice with PBS to remove all unbound genipin. All uncrosslinked (UN-XL) and crosslinked (XL) membranes were stored at 4 °C in PBS solution until analysis.

### 2.4. Biodegradation Assay with Trypsin

The effect of crosslinking on the fibrin clot and fibrin-incorporated electrospun membrane was assayed by trypsin degradation. Six samples of the fibrin clot alone and six samples of the fibrin-incorporated electrospun membranes were created from each of the centrifugation conditions at 400 g, 1450 g and 3000 g. Half of the samples were later cross-linked with 1% genipin for 48 h, as previously described. Following crosslinking, samples were placed in six well culture plates, mixed with 500 μL of 0.01% trypsin (Gibco) and incubated at 37 °C for 48 h. All samples were individually weighed prior to the assay, and measured again at 48 h.

### 2.5. Uniaxial Tensile Testing

The mechanical properties of the electrospun membrane alone (S), uncrosslinked fibrin clots (UN-XL-F), crosslinked fibrin clots (XL-F), uncrosslinked fibrin-incoporated electrospun membrane (UN-XL-S) and crosslinked fibrin-incorporated membranes (XL-S) were analyzed by uniaxial tensile testing. Preparation for uniaxial testing involved determining the thickness of each membrane and cutting it into “dog bone” specimens measuring 2.75 mm wide at their narrowest point with a length of 7.5 mm. Each specimen was then mounted onto the MTS Bionix 200 testing system (MTS Systems Corp) and stretched at rate of 10.0mm/min. Elastic modulus, energy to break and strain at break were calculated by MTS software TestWorks 4.0 and recorded. A total of six samples (n = 6) were tested for each type of membrane and all samples were tested “wet” hydrated state.

### 2.6. Statistical Analyses

Multi-way ANOVA models were used to assess the relationship between various measures (degradation, modulus, strain at break, energy to break) based on the presence or absence of the membrane, crosslinking, and centrifuge speed. A three-way interaction was fit to allow for differences based on the combination. Post-hoc pairwise comparisons were performed to determine where there were differences in materials combinations. A conservative Tukey’s HSD adjustment was used to account for multiple comparisons. A significance level of 0.05 and SAS EG v6.1 was used for all analyses.

Briefly, in models of interaction, the relationship between each independent variable and the dependent variable is called the main effect. Interaction effects occur when the effect of one variable depends on the value of another variable i.e., a third variable influences the relationship between an independent and dependent variable. In the presence of statistical significant interaction effects, it is important not to interpret the main effects without considering the interactions.

## 3. Results

### 3.1. Electrospinning PCL Membranes

**Figure 1:**
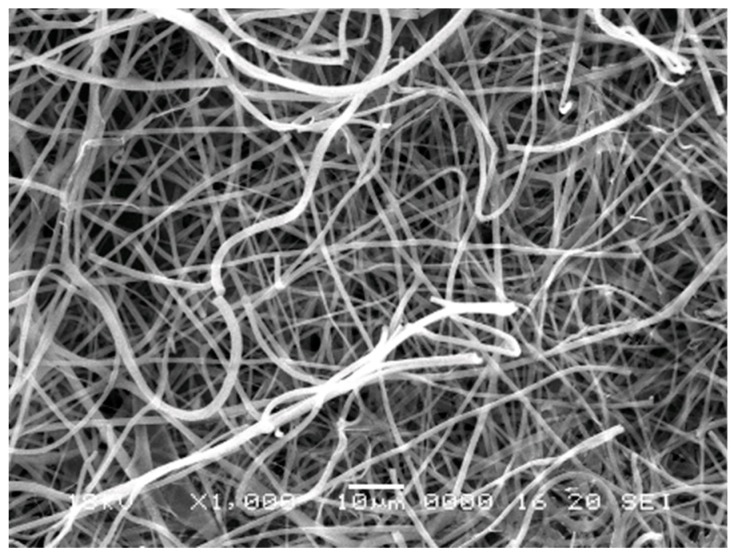
Scanning electron micrograph of electrospun polycaprolactone (PCL) membrane showing its porous and fibrous structure. The average fiber diameter is 0.82 microns.

Porous, nanofibrous membranes were generated after the optimization of electrospinning conditions. SEM analyses revealed that the average fiber diameter was 0.82 μm. There was a broad distribution of fibers with fiber-diameters ranging from 136 to 2100 nm (Figure 1).

### 3.2. Generation of Fibrin Matrix and Hybrid PCL-fibrin Matrix:

**Figure 2:**
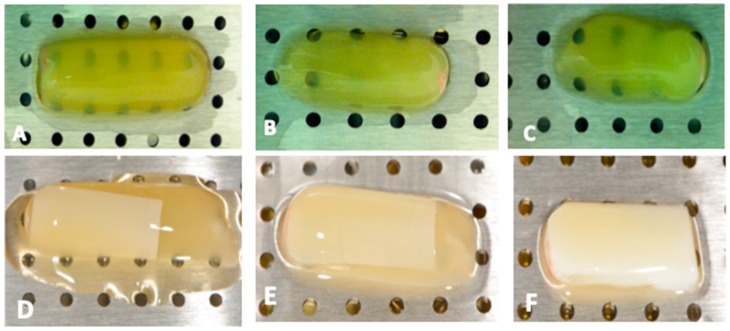
Fibrin alone (top row) and hybrid PCL-fibrin membranes (bottom row) immediately after generation following 3 centrifugation protocols: (**A**,**D**) 400 g for 12 min; (**B**,**E**) 1350 g for 15 min; (**C**,**F**) 3000 g for 60 min. While fibrin matrices are uniform, electrospun membranes within hybrid membranes can be seen as rectangular opaque sheets embedded within the gelatinous fibrin coagulum. These membranes would be compressed in a platelet-rich fibrin (PRF-box to remove excess fluid before use in experiments.

Working parameters were optimized for successful generation of fibrin matrices as well as hybrid membranes. In order to simulate platelet rich fibrin, the membranes were generated by centrifugation in uncoated glass vials and the plasma was centrifuged immediately after reconstitution with calcium chloride. Subsequent compression allowed generation of samples of uniform dimensions for testing (Figure 2).

### 3.3. Genipin Crosslinking and Trypsin Degradation Assay

**Figure 3:**
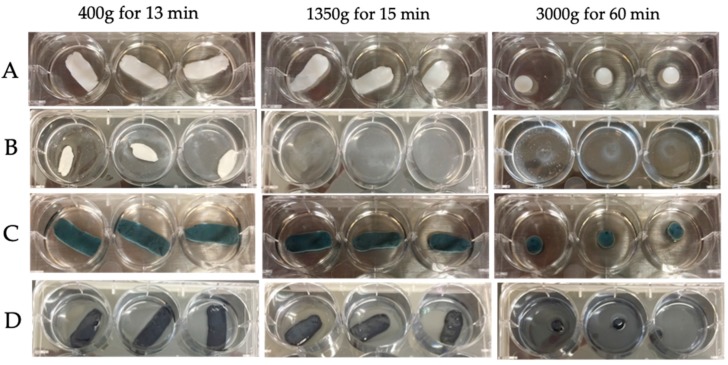
Genipin crosslinking of hybrid membranes increases biological stability following trypsin incubation. Columns indicate membranes generated by three different centrifugation protocols. Uncrosslinked fibrin matrices before (**A**) and at 48 h (**B**) showing significant structural loss at two days, especially those at 1350 and 3000 g. Crosslinked hybrid membranes before (**C**) and after (**D**) two days incubation in trypsin solution show preservation of structure especially those at 400 g and 1350 g. Membranes generated at high centrifugal forces showed less integrity to begin with.

**Figure 4:**
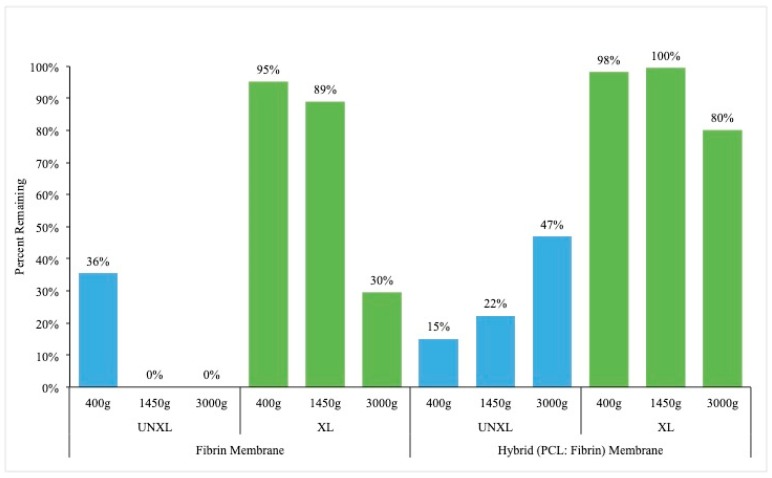
Effect of crosslinking on stability of membranes. Uncrossed fibrin matrices are completely disintegrated in trypsin with the exception of those generated at 400 g. Hybrid membranes are more resistant to trypsin degradation. However, all crosslinked membranes show remarkable stability in trypsin degradation model. Key: UNXL: uncrosslinked; XL: crosslinked.

All membranes were successfully crosslinked when treated with 1% genipin for 48 h as indicated by the blue discoloration of the membranes. Results from ANOVA model of the percent of the sample remaining after 48 h degradation with trypsin demonstrated a significant three-way interaction for the presence of a membrane, cross-linking, and the centrifuge rate (*p* = 0.0064). The highest percent remaining was seen with crosslinked samples with membrane when spun at 1450 g, though this combination was not significantly different from that at 400 g, or the samples with no membrane when spun at 400 g or 1450 g and crosslinked. The general trend was that increased centrifuge rates increased the degradation, cross-linking greatly increased the percent remaining, and the addition of the membrane provides marginal, but not significant increased stability. Estimated percent remaining is presented in Figure 3 and Figure 4. All pairwise comparisons of interest are presented in Table A4.

### 3.4. Mechanical Properties

#### 3.4.1. Baseline PCL Membrane

Results from the uniaxial tensile strength testing (n = 6) of the electrospun PCL membrane reported a mean modulus value of 47 MPa. The strain at break was 4.1 MPa and the energy to break was 100.7 N*mm.

#### 3.4.2. Modulus

**Figure 5:**
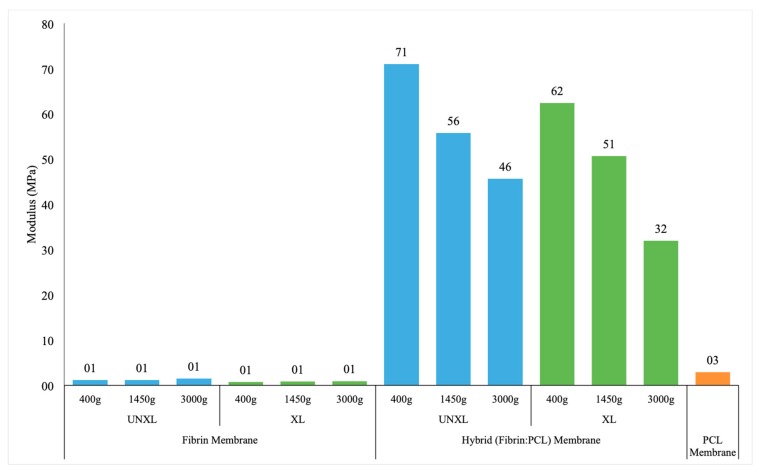
Elastic modulus of fibrin membrane, hybrid membrane and pure PCL membrane. Data shows extremely fragile fibrin matrices when compared the electrospun PCL membrane. Hybrid membrane shows improved stiffness compared to PCL indicating the additive effect of fibrin crosslinking on the electrospun PCL membrane. Key: UNXL: uncrosslinked; XL: crosslinked.

Overall, modulus was greatly increased with the presence of the PCL membrane (average 52.9 vs. 1.1 MPa). The effect of crosslinking was marginal but not statistically significant (0.0969). Figure 5 presents the estimated modulus for each sample combination. In the fibrin matrix group alone, cross-linking had little effect on the modulus; meanwhile, in the fibrin-PCL membrane group, the modulus is significantly higher compared to PCL membrane alone (47.1 MPa) which means that this hybrid membrane is stiffer and can withstand more load.

There was a significant interaction between the presence of the membrane and the centrifuge rate when modeling modulus (*p* = 0.0007). Specifically, there were no significant differences in modulus based on centrifuge rate for fibrin matrix alone, but with fibrin + membrane, as the centrifuge rate increased the modulus decreased. There was a statistically significantly higher modulus for samples with membrane when spun at 400 g vs. 3000 g (*p* < 0.0001). Pairwise comparisons by membrane and centrifuge rate are presented in Table A3. It is possible that a significant increase in the centrifugation force affected the fibrin polymerization process and could have contributed to a decrease in modulus.

#### 3.4.3. Energy to Break

**Figure 6:**
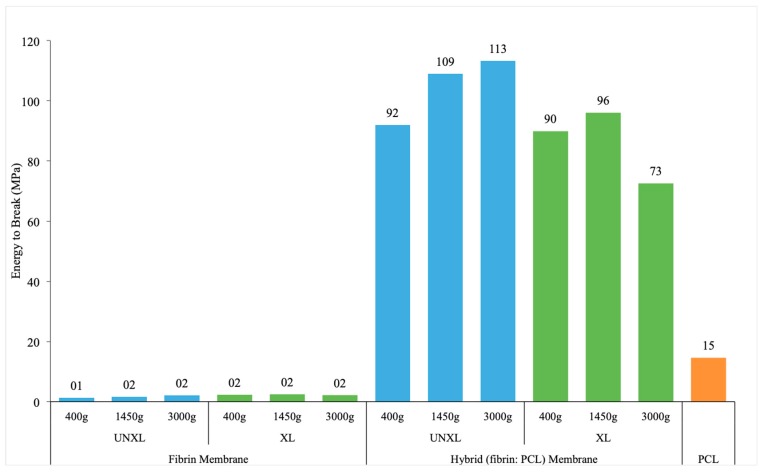
Energy to break of fibrin membrane, hybrid membrane and pure PCL membrane. Data shows extremely fragile fibrin matrices when compared the electrospun PCL membrane. Hybrid membrane shows improved stiffness compared to PCL indicating the additive effect of fibrin crosslinking on the electrospun PCL membrane. Key: UNXL: uncrosslinked; XL: crosslinked.

The only statistically significant predictor of energy to break was the presence or absence of a membrane (*p* < 0.0001) (Table A4). The presence of a membrane increased the energy to break by over 90 N*mm (95% CI: 80.8–103.2). After adjusting for membrane, the effects for the crosslinking and centrifuge rate were not significantly different. Estimated mean energy to break for different configurations are presented in Figure 6.

#### 3.4.4. Strain at Break

**Figure 7:**
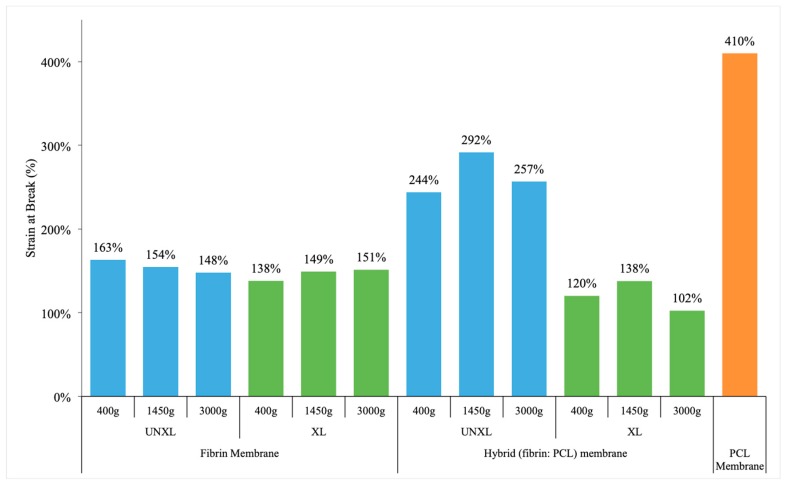
Strain at break of fibrin membrane, hybrid membrane and pure PCL membrane. Data shows fibrin membranes stretch approximately 50% of its original length. Hybrid membranes, especially those that are not crosslinked have significantly increased strain before break (more than twice its original length). Crosslinking decreases the ability of the membranes to stretch as expected. Key: UNXL: uncrosslinked; XL: crosslinked.

The strain at break essentially measures how much the material elongates before it breaks. It is the ratio of change in length at break to original length and is often represented as a percentage. Figure 7 indicates a significant effect of crosslinking on the strain at break only in the presence of a scaffold and the effect of crosslinking was negligible in the fibrin matrix alone group. However, when we look at the group with the scaffold, crosslinking of the fibrin-incorporated matrix actually reduces the strain at break to a level similar to the fibrin matrix alone group. In a practical setting, this means a decrease in the ability of the material to be stretched once it is crosslinked. The greatest strain at break is seen in the uncrosslinked sample with scaffold at 1450 g, but that is not statistically significantly different from that at 400 g or 3000 g. One possible explanation is that crosslinking can result in significant dehydration making the material stiffer and reduces ability to be stretched.

The effect of crosslinking on strain at break was different based on the presence of a membrane (*p* < 0.0001) (Table A5). Centrifuge speed was not significantly associated with a change in strain at break (*p* = 0.2116). The effect of crosslinking was negligible for samples with no membrane (*p* = 0.9116), but significant in presence of membrane. For samples with membrane, crosslinking significantly decreased the strain at break by an average of 1.44 (*p* < 0.0001). The greatest strain at break was seen in un-crosslinked samples with a membrane, spun at 1450 g, but this was not significantly different from samples at either of the other two centrifuge rates (400 or 3000 g).

## 4. Discussion

The use of a barrier membrane in GTR also plays an important role in space maintenance and stabilization of the clot in the initial phases of wound healing [26]. Two main types of barrier membranes are available on the market: resorbable and non-resorbable. While non-resorbable membranes guarantee space maintenance for the duration of healing, it requires a second surgery for their removal; complications have also been reported such as infection during membrane exposures that can negatively influence clinical outcomes of regenerative procedures [27]. The resorbable membranes are derived from bovine or porcine Type I collagen, and have the advantage of being recognized by the body benefits of biologic membranes i.e., can readily bind to cell surface receptors to facilitate wound healing. In vivo studies have demonstrated that collagen materials can positively influence chemotaxis of periodontal ligament fibroblasts [28] and gingival fibroblasts [29] to effect repair of damaged tissues. Although the biodegradable nature of biologic membranes eliminates the need for surgical membrane retrieval, these membranes collagen membranes present limitations in terms of controlling degradation and mechanical strength [30].

In this study, our objective was to optimize the centrifugation conditions to generate a hybrid membrane by incorporating fibrin matrix into an electrospun membrane. PRF has been a biomaterial of interest to our lab and we have previously characterized its mechanical properties [31]. The goal of the current study is to combine our expertise in electrospinning with the biologically attractive analog of platelet rich fibrin to generate the hybrid membrane. PRF is a term strictly restricted to refer to an autologous platelet concentrate generated by centrifugation of whole blood in a glass vial without any addition of calcium or thrombin. Even though the method of generating fibrin matrix in this study may closely resemble the protocol for PRF, there are some important differences: Unlike fresh, whole blood with no anticoagulants used in PRF, we use never-frozen, fresh pooled human plasma collected in citrate. In order to preserve the function of platelets, the plasma were used within 4 days of collection for all our experiments.

Despite the popularity and increased clinical use of autologous platelet concentrates (esp. platelet rich plasma), there are currently no standardized methods of preparation. Most preparations involve two spins of the whole blood in a centrifuge: a soft spin to remove the red blood cells and a hard spin to concentrate the platelets [32]. In our study, we used commercially available pooled human plasma that had already undergone soft spin to remove the red cells. The plasma was then subjected to three different protocols for the hard spin to concentrate platelets: 400 g for 12 min; 1450 g for 15 min; and 3000 g for 60 min. 400 g for 12 min was selected as the benchmark since this is the centrifugation protocol currently used for the creation of L-PRF through the intra-spin system (intra-lock) [33]; 3000 g for 1 h was selected as the upper limit as this was the greatest speed permitted on the centrifuge. Lastly, 1450 g was selected as the relative halfway mark between 400 g and 3000 g. It was hypothesized that the membranes created at 3000 g would exhibit enhanced mechanical properties due to greater condensation of the fibrin matrix at an increased centrifugation force.

Based on the results from the uniaxial mechanical test, the mechanical properties of the fibrin-incorporated electrospun membrane exhibited similar characteristics at 400 g and 1450 g with no statistical significant difference in the modulus, energy to break or strain at break. The modulus describes the elastic properties of a biomaterial and is a measure of its stiffness. Our study reported that an increase in the centrifugation speed negatively altered the stiffness of the membrane. It is possible that a significant increase in the centrifugation force did not allow the fibrin matrix to properly engage with the nanostructure of the electrospun membrane during the polymerization process. Rather, the fibrin matrix was simply layered on top of the membrane and thus membranes created at 3000 g lacked the inherent stiffness of the membrane and could explain the observed decrease in modulus.

The stability of the novel biomaterial was also examined through the effects of crosslinking. Crosslinking is the process of chemically joining two or more molecules together with the purpose of stabilizing the protein structure [34]. Our study showed a general trend that crosslinking significantly increased the stability of both the fibrin clots and fibrin-incorporated electrospun membranes. This is in agreement with more recent findings that addition of genipin significantly improved mechanical properties of fibrin hydrogels by increasing the compressive, tensile, and shear moduli [35]. While the membrane provided a marginal (not significant) increase in stability in the presence of crosslinking, its effect was significant if fibrin was not crosslinked.

## 5. Conclusions

Our results showed that the combined presence of both the fibrin matrix and electrospun membrane led to statistically significant improvements in mechanical properties. Meanwhile, crosslinking enhanced the biologic stability of the fibrin matrix as evidenced by a greater resistance against enzymatic degradation following trypsin exposure. Lastly, centrifugation speeds at 400 g and 1450 g produced membranes exhibiting similar mechanical properties. However, at 3000 g the mechanical properties were negatively influenced by an increase in the centrifugation speed. Based on the results of this study, crosslinked electrospun PCL with fibrin-incorporated membrane generated at 400 g exhibits superior biologic stability and mechanical properties. Overall, a hybrid barrier membrane that contains biologically active fibrin matrix into a synthetic polymeric electrospun membrane has great potential as a novel biomaterial in periodontal surgery.

## Appendix A

**Table A1:** ANOVA model results (* Statistically significant at 0.05 level).

	Trypsin Degradation	Modulus	Strain at Break	Energy to Break
Membrane (yes/no)	<0.0001 *	<0.0001 *	<0.0001 *	<0.0001 *
Centrifuge Rate (400, 1450, 3000)	<0.0001 *	0.0008 *	0.2116	0.5871
Crosslinking (XL or UNXL)	<0.0001 *	0.0969	<0.0001 *	0.0736
Membrane × centrifuge rate	<0.0001 *	0.0007 *	0.2777	0.5974
Centrifuge rate × crosslinking	<0.0001 *	0.8140	0.9735	0.2512
Membrane × crosslinking	0.2290	0.1307	<0.0001 *	0.0544
Membrane × centrifuge rate × crosslinking	0.0064 *	0.8352	0.4000	0.2831

**Table A2:** Pairwise comparisons of % remaining for membrane, crosslinking and centrifugation rates. * indicates statistical significance (*p* < 0.05).

Comparison	Estimated Difference (% Remaining)	Adj P
No membrane at 400 g: UNXL vs. XL	−60%	<0.0001 *
No membrane at 1450 g: UNXL vs. XL	−89%	<0.0001 *
No membrane at 3000 g: UNXL vs. XL	−30%	<0.0001 *
Membrane at 400 g: UNXL vs. XL	−83%	<0.0001 *
Membrane at 1450 g: UNXL vs. XL	−77%	<0.0001 *
Membrane at 3000 g: UNXL vs. XL	−33%	<0.0001 *
No membrane UNXL: 400 g vs. 1450 g	36%	<0.0001 *
No membrane UNXL: 400 g vs. 3000 g	36%	<0.0001 *
No membrane UNXL: 1450 g vs. 3000 g	0%	1
No membrane XL: 400 g vs. 1450 g	6%	0.9887
No membrane XL: 400 g vs. 3000 g	66%	<0.0001 *
No membrane XL: 1450 g vs. 3000 g	59%	<0.0001 *
Membrane UNXL: 400 g vs. 1450 g	−7%	0.9637
Membrane UNXL: 400 g vs. 3000 g	−32%	<0.0001 *
Membrane UNXL: 1450 g vs. 300 g	−25%	0.0001 *
Membrane XL: 400 g vs. 1450 g	−1%	1
Membrane XL: 400 g vs. 3000 g	18%	0.0235 *
Membrane XL: 1450 g vs. 3000 g	19%	0.0096 *
UNXL at 400 g: no membrane vs. membrane	20%	0.0044 *
UNXL at 1450 g: no membrane vs. membrane	−22%	0.001 *
UNXL at 3000 g: no membrane vs. membrane	−47%	<0.0001 *
XL at 400 g: no membrane vs. membrane	−3%	1
XL at 1450 g: no membrane vs. membrane	−11%	0.6658
XL at 3000 g: no membrane vs. membrane	−51%	<0.0001 *

**Table A3:** Comparison of mean modulus by membrane and centrifuge rates.

Comparison	Estimated Difference (MPa)	Adj P
400 g: No membrane vs. membrane	−65.70	<0.0001
1450 g: No membrane vs. membrane	−52.21	<0.0001
3000 g: No membrane vs. membrane	−37.60	<0.0001
No membrane: 1450 g vs. 3000 g	−0.20	1
No membrane: 400 g vs. 1450 g	−0.01	1
No membrane: 400 g vs. 3000g	−0.21	1
Membrane: 1450 g vs. 3000 g	1,5.41	0.0403
Membrane: 400 g vs. 1450 g	13.47	0.0600
Membrane: 400 g vs. 3000 g	27.88	<0.0001

**Table A4:** Estimated mean energy to break for membrane (yes/no).

Membrane	Estimated Mean (N*mm)	SE	*p*-Value *
No membrane	2.0	3.55	
Membrane	95.5	3.45	
Difference (no membrane−membrane)	−93.5	4.95	<0.0001

* *p*-value for t-test of difference in means.

**Table A5:** Comparison of mean strain at break by membrane and crosslinking. * indicates statistical significance (*p* < 0.05).

Comparison	Estimated Difference (mm/mm)	Adj P
UNXL: no membrane vs. membrane	−1.09	<0.0001 *
No membrane: UNXL vs. XL	0.09	0.9116
Membrane: UNXL vs. XL	1.44	<0.0001 *
XL: no membrane vs. membrane	0.26	0.2193
